# Direct Angiotensin II Type 2 Receptor Stimulation Ameliorates Insulin Resistance in Type 2 Diabetes Mice with PPARγ Activation

**DOI:** 10.1371/journal.pone.0048387

**Published:** 2012-11-14

**Authors:** Kousei Ohshima, Masaki Mogi, Fei Jing, Jun Iwanami, Kana Tsukuda, Li-Juan Min, Akiyoshi Ogimoto, Björn Dahlöf, Ulrike M. Steckelings, Tomas Unger, Jitsuo Higaki, Masatsugu Horiuchi

**Affiliations:** 1 Department of Molecular Cardiovascular Biology and Pharmacology, Ehime University, Graduate School of Medicine, Tohon, Ehime, Japan; 2 Department of Integrated Medicine and Informatics, Ehime University, Graduate School of Medicine, Tohon, Ehime, Japan; 3 Department of Medicine, Sahlgrenska University Hospital/Östra, University of Gothenburg, Gothenburg, Sweden; 4 Center for Cardiovascular Research, Institute of Pharmacology, Charité University Medicine, Berlin, Germany; 5 CARIM - School for Cardiovascular Diseases, Maastricht University, Maastricht, The Netherlands; Warren Alpert Medical School of Brown University, United States of America

## Abstract

**Objectives:**

The role of angiotensin II type 2 (AT_2_) receptor stimulation in the pathogenesis of insulin resistance is still unclear. Therefore we examined the possibility that direct AT_2_ receptor stimulation by compound 21 (C21) might contribute to possible insulin-sensitizing/anti-diabetic effects in type 2 diabetes (T2DM) with PPARγ activation, mainly focusing on adipose tissue.

**Methods:**

T2DM mice, KK-Ay, were subjected to intraperitoneal injection of C21 and/or a PPARγ antagonist, GW9662 in drinking water for 2 weeks. Insulin resistance was evaluated by oral glucose tolerance test, insulin tolerance test, and uptake of 2-[^3^H] deoxy-D-glucose in white adipose tissue. Morphological changes of adipose tissues as well as adipocyte differentiation and inflammatory response were examined.

**Results:**

Treatment with C21 ameliorated insulin resistance in KK-Ay mice without influencing blood pressure, at least partially through effects on the PPARγ pathway. C21 treatment increased serum adiponectin concentration and decreased TNF-α concentration; however, these effects were attenuated by PPARγ blockade by co-treatment with GW9662. Moreover, we observed that administration of C21 enhanced adipocyte differentiation and PPARγ DNA-binding activity, with a decrease in inflammation in white adipose tissue, whereas these effects of C21 were attenuated by co-treatment with GW9662. We also observed that administration of C21 restored β cell damage in diabetic pancreatic tissue.

**Conclusion:**

The present study demonstrated that direct AT_2_ receptor stimulation by C21 accompanied with PPARγ activation ameliorated insulin resistance in T2DM mice, at least partially due to improvement of adipocyte dysfunction and protection of pancreatic β cells.

## Introduction

The angiotensin (Ang) II type 1 (AT_1_) receptor mediates the major effects of Ang II in the pathogenesis of insulin resistance and subsequent type 2 diabetes mellitus (T2DM) [1]. AT_1_ receptor blockers (ARBs) are known to improve insulin resistance and reduce the new onset of diabetes [2]–[6]. When the AT_1_ receptor is blocked by ARBs and unbound Ang II can act on the Ang II type 2 (AT_2_) receptor, stimulation of the AT_2_ receptor might be involved in the effects of ARBs. AT_2_ receptor stimulation appears to antagonize the signaling activated by AT_1_ receptor stimulation in various tissues [7]; however, the role of AT_2_ receptor stimulation in metabolic disorders is still unclear. We demonstrated that there was no apparent difference in insulin-mediated glucose uptake into skeletal muscle between wild-type and AT_2_ receptor null mice, whereas insulin-induced glucose uptake in white adipose tissue in AT_2_ receptor null mice was significantly lower than that of control mice.^3^ It was reported that AT_2_ receptor-dependent Ang II signaling increases adipose cell mass and glucose intolerance, thereby participating in the deleterious effects of a high-fat diet [8]. Mitsuishi et al demonstrated that the Ang II-induced reduction in muscle mitochondria in mice was partially, but significantly, reversed by blockade of either the AT_1_ receptor or AT_2_ receptor, associated with increased fat oxidation, decreased muscle triglyceride, and improved glucose tolerance [9]. In terms of mitochondria, Abadir et al recently presented evidence of age-related changes in mitochondrial Ang II receptor expression, i.e., increased mitochondrial AT_1_ receptor and decreased AT_2_ receptor density, which was reversed by chronic treatment with an ARB, and demonstrated that activation of the mitochondrial AT_2_ receptor increased NO production and probably attenuated aging [10]. The availability of selective agonists of the AT_2_ receptor could be useful in helping to resolve some of the apparently conflicting results regarding the role of the AT_2_ receptor in various pathological conditions. The recent development of selective non-peptidic AT_2_ receptor agonists has provided new important tools for further evaluation of the roles of AT_2_ receptor stimulation in pathophysiological conditions and should relaunch interest in studying the intracellular effects and regulation of this receptor in various tissues [11], [12].

Adipose tissue as an endocrine organ plays a crucial role in the pathogenesis of insulin resistance and the onset of type 2 diabetes. Adipose tissue contains renin, angiotensinogen and angiotensin-converting enzyme (ACE), which results in increased production of Ang II as a local regulator of adipose tissue functions. Previous reports indicated that blockade of AT_1_ receptor stimulation attenuated adipocyte dysfunction; however, the effects of AT_2_ receptor stimulation on adipose tissue functions are not yet clear. We reported that the weight of both epididymal and retroperitoneal adipose tissue in ApoE knockout (KO) mice with AT_2_ receptor deletion (AT_2_/ApoEKO) was greater than that in ApoEKO mice after a high-cholesterol diet. In adipose tissue of AT_2_/ApoEKO mice, the adipocyte number was decreased and the expression of peroxisome proliferator-activated receptor gamma (PPARγ), CCAAT-enhancer-binding protein α (C/EBPα, and adipocyte Protein 2 (aP2) was lower than that in ApoEKO mice, in association with an increase in nicotinamide adenine dinucleotide phosphate (NADPH) oxidase activity, suggesting that AT_2_ receptor stimulation in adipose tissue is involved in the improvement of adipocyte differentiation and adipose tissue dysfunction in an atherosclerotic model [13]. It was previously reported that the AT_2_ receptor also mediates the effect of Ang II to induce the production and release of prostacyclin from adipocytes, which in turn stimulates differentiation of adipose precursor cells [14], [15]. Yvan-Charvet L et al also reported that adipocyte hypertrophy and increased lipogenic gene expression induced by adipose angiotensinogen overproduction was prevented by deletion of the AT_2_ receptor [16]. These results led us to examine the roles of direct AT_2_ receptor stimulation by compound 21 (C21) in adipose tissue in terms of glucose intolerance using type 2 diabetic mice KK-Ay.

PPARγ, a nuclear transcription factor, plays an important role in regulation of adipocyte differentiation, insulin resistance, and inflammation [17]. In addition, large clinical studies have demonstrated that a PPARγ agonist had beneficial effects on not only glycemic control but also preventing atherosclerotic disease [18]–[21]. Possible crosstalk between PPARγ and the AT_1_ receptor such as a decrease in AT_1_ receptor promoter activity and AT_1_ receptor expression by PPARγ activation has been suggested [22], [23]. It has also been reported that AT_1_ receptor blockade decreases NF-κB activation, with PPARγ activation in the vasculature [24]. AT_1_ receptor stimulation activates ERK, and PPARγ stimulation inhibits this ERK activation in VSMC [25]. On the other hand, it is reported that Ang II induces PPARγ activation in PC12W cells via AT_2_ receptor activation [26]. Accordingly, we hypothesized that AT_2_ receptor stimulation by C21 might contribute to possible insulin-sensitizing/anti-diabetic effects in type 2 diabetes, with PPARγ activation. It is also clinically important to investigate the possibility that crosstalk between AT_2_ receptor stimulation and PPARγ activation may regulate adiopocyte function and insulin resistance, and therefore we also examined this possibility in this study.

## Methods

### Animal and treatment

Eight-week-old adult male KK-Ay mice (CLEA, Tokyo, Japan) were used in this study. They were housed in an air conditioned room at 25°C with a 12-h light/12-h dark cycle. They were given a standard diet (MF, Oriental Yeast, Tokyo, Japan) and water *ad libitum*. Compound 21 was provided by Vicore Pharma (Gothenburg, Sweden). KK-Ay mice were subjected to intraperitoneal injection of C21 (10 µg/kg per day) dissolved in 100 µl of phosphate buffered saline (PBS) for 2 weeks. Some KK-Ay mice treated with C21 were concurrently administered GW9662, a PPARγ antagonist, at a dose of 0.35 mg/kg per day in drinking water. The control mice were given intraperitoneal injection of an equivalent volume of vehicle as the experimental group. Systolic blood pressure (SBP) was measured by the tail-cuff method (MK-2000ST, Muromachi Kikai, Co. Ltd., Tokyo, Japan) as described previously [27]. All procedures were performed in accordance with the National Institutes of Health Guide for the Care and Use of Laboratory Animals, and reviewed and approved by the Animal Studies Committee of Ehime University.

### Oral glucose tolerance test and insulin tolerance test

Oral glucose tolerance test (OGTT) was performed after 16-hour overnight fasting [28]. Glucose solution (2 g/kg) was administered orally, and a small amount of blood was obtained from the orbital sinus or tail vein without anesthesia at 0, 30, 60 and 120 min after glucose loading. For insulin tolerance test (ITT), mice were given an intraperitoneal injection of 0.5 U/kg insulin solution (NovoRapid, Novo Nordisk, Bagsvaerd, Denmark) after 4-hour fasting. Blood glucose concentration was determined by glucose dehydrogenase pyrroloquinolinequinone method (Freestyle, Nipro, Osaka, Japan). Serum insulin concentration was measured by enzyme-linked immunosolvent assay (ELISA) (Ultra Sensitive Rat Insulin kit, Morinaga Institute of Biological Science, Kanagawa, Japan).

### Morphological analysis of adipose tissue and pancreatic tissue

Epididymal and retroperitoneal white adipose tissue was taken, fixed with formalin, and paraffin-embedded sections were prepared [29]. After staining the sections with fuchsin, adipocyte number in three microscopic fields was counted and expressed as the cell number per mm^2^. Pancreatic tissue was taken similarly, fixed with formalin, and paraffin-embedded sections were stained with Gomori's aldehyde-fuchsin to visualize β cell granules in islets [30]. The ratio of stained area (β cell area) was calculated using computer-imaging software (Densitograph; ATTO Corporation). Values were obtained from five different mice in each group.

### Measurement of serum insulin, adiponectin and TNF-α concentrations

Serum concentrations of insulin (Ultra Sensitive Rat Insulin kit, Morinaga Institute of Biological Science, Kanagawa, Japan), adiponectin (Mouse/Rat High Molecular Weight Adiponectin ELISA KIT, AKMAN-011, Shibayagi, Gunma, Japan), and tumor necrosis factor (TNF)-α (Mouse TNF-α ELISA KIT, AKMTN-011, Shibayagi, Gunma, Japan) were measured using commercially available kits.

### Measurement of rate constant of net tissue uptake of 2-[^3^H]deoxy-D-glucose

Uptake of 2-[^3^H]deoxy-D-glucose (2-[^3^H]DG) in peripheral tissues was measured as previously reported [31]. Epididymal and retroperitoneal white adipose tissue was rapidly dissected and weighed. The rate constant of net tissue uptake of 2-[^3^H]DG was calculated as described previously [32].

### Quantitative reverse-transcription polymerase chain reaction (RT-PCR)

Real-time quantitative reverse-transcription polymerase chain reaction (PCR) was performed with a SYBR Premix Ex Taq (Takara Bio Inc., Japan). mRNAs were prepared from epididymal and retroperitoneal white adipose tissue after treatment with or without C21 and/or GW9662. PCR primers were as follows: 5′-CACCCAAGGGAACTTGTGCAG-3′ (forward) and 5′-GGTCGTAGGTGAAGAGAACGG-3′ (reverse) for adiponectin, 5′-TGGAGACCGCCCAGGCTTG-3′ (forward) and 5′-GTCTGTCATCTTCTGGAGCACCTT-3′ (reverse) for PPARγ, 5′-CAAAGCCAAGAAGTCGGTGGACAA-3′ (forward) and 5′-TCATTGTGACTGGTCAACTCCAGC-3′ (reverse) for C/EBPα, 5′-AACACCGAGATTTCCTT-3′ (forward) and 5′-ACACATTCCACCACCAG-3′ (reverse) for aP2, 5′-CGAGTGACAAGCCTGTAGCC-3′ (forward) and 5′-GGTGAGGAGCACGATGTCG-3′ (reverse) for tumor necrosis factor-α (TNF-α), 5′-TTAACGCCCCACTCACCTGCTG-3′ (forward) and 5′-GCTTCTTTGGGACACCTGCTGC-3′ (reverse) for monocyte chemoattractant protein-1 (MC-1), 5′-CCACTTCACAAGTCGGAGGCTTA-3′ (forward) and 5′-GCAAGTGCATCATCGTTGTTCATAC-3′ (reverse) for interleukin-6 (IL-6), 5′-ATGTAGGCCATGAGGTCCAC-3′ (forward) and 5′-TGCGACTTCAACAGCAACTC-3′ (reverse) for glyceraldehyde-3-phosphate dehydrogenase (GAPDH).

### DNA-binding activity of PPARγ in adipose tissue

Nuclear extract from adipose tissue was prepared using a nuclear extract kit (Sigma–Aldrich, Japan) after homogenization of adipose tissue with a glass homogenizer according to the manufacturer's protocol. DNA-binding of PPARγ was determined with a DNA-binding ELISA kit (TransAM™ PPARγ, Active Motiv, Carlsbad, CA). The kit is designed to detect PPARγ protein in nuclear extract, which binds to PPARγ response element immobilized at the bottom of the 96-well plate, using specific antibodies conjugated to horseradish peroxidase. Five micrograms of protein from each nuclear extract was applied to the assay, and DNA-binding of PPARγ was determined.

### Statistical analysis

All values are expressed as mean ± standard error in the text and figures. Data were evaluated by analysis of variance (ANOVA) followed by post-hoc analysis for multiple comparisons. A difference with *p*<0.05 was considered significant.

## Results

### Effect of compound 21 on blood glucose, serum insulin, adiponectin, and TNF-α concentrations

**Figure 1:**
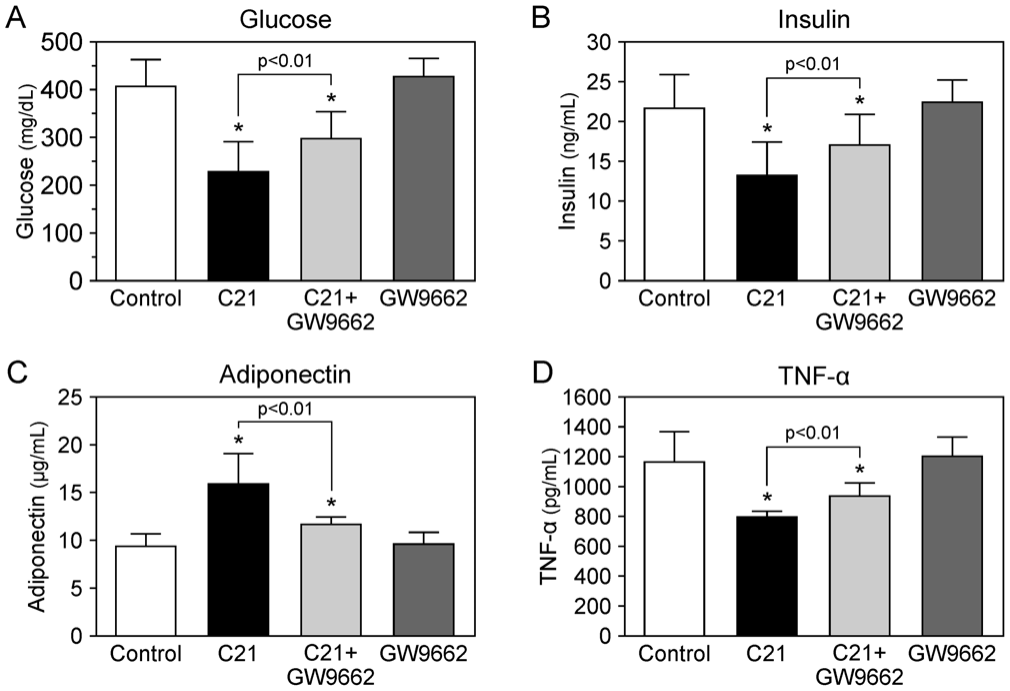
Effect of compound 21 administration on blood glucose, insulin, and adipocytokines in KK-Ay mice. Concentrations of blood glucose (**A**) and serum insulin (**B**) in KK-Ay mice treated with compound 21 (C21). KK-Ay mice were treated as described in ‘Methods’. Some KK-Ay mice treated with C21 were concurrently administered GW9662, a PPARγ antagonist. Blood samples were obtained under a fed condition. Effect of compound 21 on concentrations of adiponectin (**C**) and TNF-α (**D**) in KK-Ay mice. Blood samples were obtained after 16 hours of fasting. Concentrations of serum adiponectin and TNF-α in KK-Ay mice were measured by ELISA. Data are expressed as mean ± SEM. n = 10 for each group. *p<0.01 vs. control.

Treatment of KK-Ay with an AT_2_ receptor agonist, C21 (10 µg/kg per day), for 14 days did not change systolic blood pressure compared with that in control KK-Ay mice. (data not shown) The increase in body weight of KK-Ay was attenuated with C21 treatment, whereas this effect of C21 was attenuated by GW9662 administration (**Table 1** **and** **2**). Food intake was not different in each group. Treatment with C21 significantly reduced blood glucose concentration and serum insulin concentration in the fed condition compared with those in control KK-Ay mice (**Figure 1A and 1B**). The decreases of blood glucose and serum insulin concentrations by C21 treatment were attenuated by co-administration of GW9662, whereas treatment with GW9662 alone did not affect blood glucose and serum insulin concentrations. We also observed that C21 treatment increased serum adiponectin concentration, but decreased serum TNF-α concentration compared with those in control KK-Ay mice (**Figure 1C and 1D**). GW9662 treatment attenuated the C21-mediated increase of serum adiponectin concentration and the decrease of serum TNF-α concentration, whereas treatment with GW9662 alone did not affect them.

**Table 1:** Effect of compound 21 on body weight in KK-Ay mice.

	Body weight (g)		
Group	Baseline	Day 7	Day 14
Control	38.6±2.2	39.7±2.6	41.0±2.8
C21	38.3±1.7	38.7±1.1* †	39.3±1.1* † ‡
C21+GW9662	38.6±1.4	39.1±1.6	39.9±0.7*
GW9662	38.5±1.9	39.5±2.2	41.3±2.5

Animals were fed normal standard diet for 2 weeks. Values are mean±SEM of 15 to 20 mice for each group. C21, compound 21.

* *p*<0.01 vs. control;

† *p*<0.01 vs. GW9662;

‡ *p*<0.01 vs. C21+GW9662.

**Table 2:** Effect of compound 21 on food intake in KK-Ay mice.

Group	Food intake (g/day)
Control	6.7±0.7
C21	6.4±0.6
C21+GW9662	6.3±0.7
GW9662	6.5±0.6

Animals were fed normal standard diet for 2 weeks. Values are mean±SEM of 15 to 20 mice for each group. C21, compound 21.

### Effect of compound 21 on glucose intolerance and insulin sensitivity

**Figure 2:**
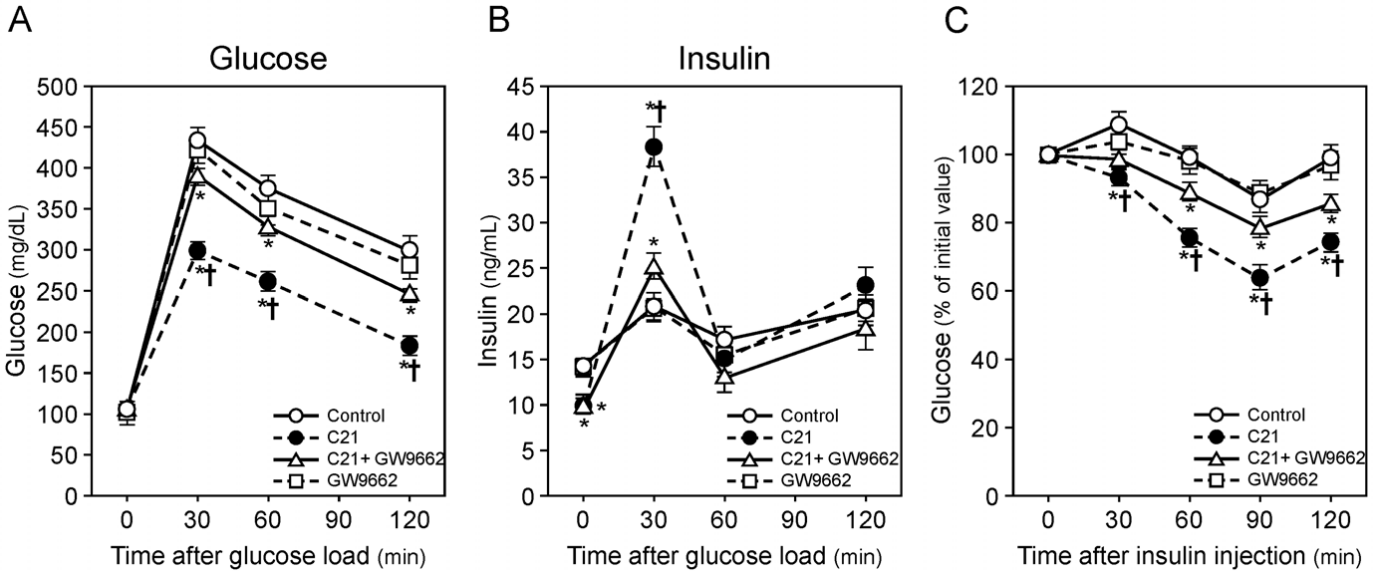
Effect of compound 21 administration on glucose intolerance in KK-Ay mice. Effect of administration of compound 21 (C21) on concentrations of blood glucose (**A**) and serum insulin (**B**) in oral glucose tolerance test (OGTT), and glucose concentration in insulin tolerance test (ITT) (**C**) in KK-Ay mice. Male KK-Ay mice were treated as described in ‘Methods’. Some KK-Ay mice treated with C21 were concurrently administered GW9662, a PPARγ antagonist. OGTT and ITT were performed after 16 hours and 4 hours of fasting, respectively (n = 8–10 per group). Data are expressed as mean ± SEM. *p<0.01 vs. control; †*p*<0.01 C21 vs. C21+GW9662.

In OGTT, the basal blood glucose concentration after 16-hour fasting did not differ between each group, whereas the peak of the glucose rise in response to a glucose load was lower and the decrease of blood glucose concentration was faster in mice treated with C21 compared with control and GW9662-treated KK-Ay mice (**Figure 2A**). Serum insulin concentration was lower in mice treated with C21 compared with control and GW9662-treated mice. Moreover, we observed that C21 treatment increased serum insulin concentration at 30 minutes after a glucose load (**Figure 2B**). The decrease of blood glucose concentration and the increase of serum insulin concentration after a glucose load by C21 treatment were attenuated by GW9662 administration (**Figure 2A and 2B**). In ITT, the decrease of blood glucose concentration by insulin injection was further enhanced in the C21-treated group, while this increase of insulin response by C21 treatment was inhibited by GW9662 treatment (**Figure 2C**).

### Effect of compound 21 on glucose uptake in adipose tissue of KK-Ay mice

**Figure 3:**
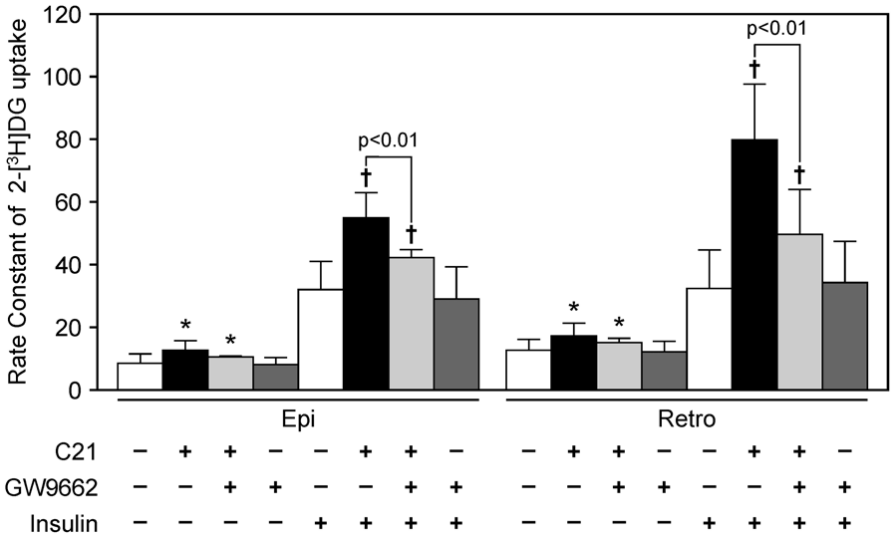
Effect of compound 21 administration on glucose uptake in KK-Ay mice. Rate constant of 2-[^3^H]deoxy-D-glucose (2-[^3^H]DG) uptake in epididymal (Epi) and retroperitoneal (Retro) white adipose tissue. Rate constant of 2-[^3^H]DG uptake was determined with or without insulin (1.0 U/kg) injection as described in “Methods” (n = 6–8 per group). Data are expressed as mean ± SEM. *p<0.01 vs. control (insulin−); †*p*<0.01 vs. control (insulin+). C21, compound 21.

Administration of C21 significantly increased 2-[^3^H]DG uptake in white adipose tissue with or without insulin stimulation (**Figure 3**). The enhancement of 2-[^3^H]DG uptake after insulin injection by C21 was attenuated by co-administration of GW9662 in white adipose tissue, whereas treatment with GW9662 alone did not affect 2-[^3^H]DG uptake.

### Adipose tissue weight and adipocyte size after treatment with compound 21

**Figure 4:**
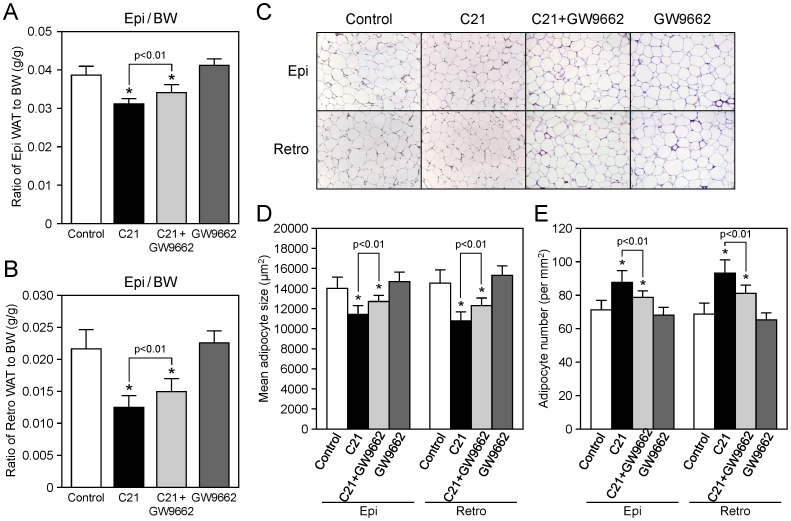
Effect of compound 21 administration on adipose tissue weight and adipocyte size in KK-Ay mice. KK-Ay mice were treated as described in ‘Methods’. Ratio of white adipose tissue (WAT) weight to body weight (BW) in epididymal (Epi) WAT (**A**) and retroperitoneal WAT (Retro) (**B**) (n = 10 for each group). Representative microscopic photos of adipose tissue (**C**) and measurement of adipocyte size (**D**) and Mean adipocyte number (**E**) in KK-Ay mice. Formalin-fixed, paraffin-embedded sections of Epi and Retro white adipose tissue were stained by fuchsin. Adipocyte size was measured in three microscopic fields (40–60 adipocytes per field) for each section using computer-imaging software. Data are expressed as mean ± SEM. n = 5 to 6 per group. *p<0.01 vs. control. C21, compound 21.

To examine the possible mechanism of the C21-mediated improvement of glucose intolerance in KK-Ay mice, we focused on adipose tissue. Treatment with C21 for 14 days significantly decreased the ratio of epididymal and retroperitoneal adipose tissue weight to body weight, whereas this ratio became higher with GW9662 co-treatment (**Table 3** **,** **Figure 4A and 4B**). On histological analysis, mean adipocyte size in white adipose tissue was smaller in the C21-treated group, and consequently the number of adipocytes was increased by C21 treatment (**Figure 4C, 4D and 4E**). The C21-mediated decrease in adipocyte size and increase in adipocyte number were attenuated by GW9662 administration, while GW9662 treatment alone did not influence adipocyte size or number.

**Table 3:** Effect of compound 21 on adipose tissue weight in KK-Ay mice.

	Adipose tissue weight (g)	
Group	Epi	Retro
Control	1.60±0.10	0.89±0.12
C21	1.22±0.06* † ‡	0.49±0.07* † ‡
C21+GW9662	1.36±0.08* †	0.60±0.08* †
GW9662	1.69±0.07	0.93±0.08

Values are mean±SEM of 10 mice for each group. C21, compound 21; Epi, epididymal; Retro, retroperitoneal.

* *p*<0.01 vs. control;

† *p*<0.01 vs. GW9662;

‡ *p*<0.01 vs. C21+GW9662.

### Effect of compound 21 on expression of adipocyte differentiation markers and DNA-binding activity of PPARγ in white adipose tissue

**Figure 5:**
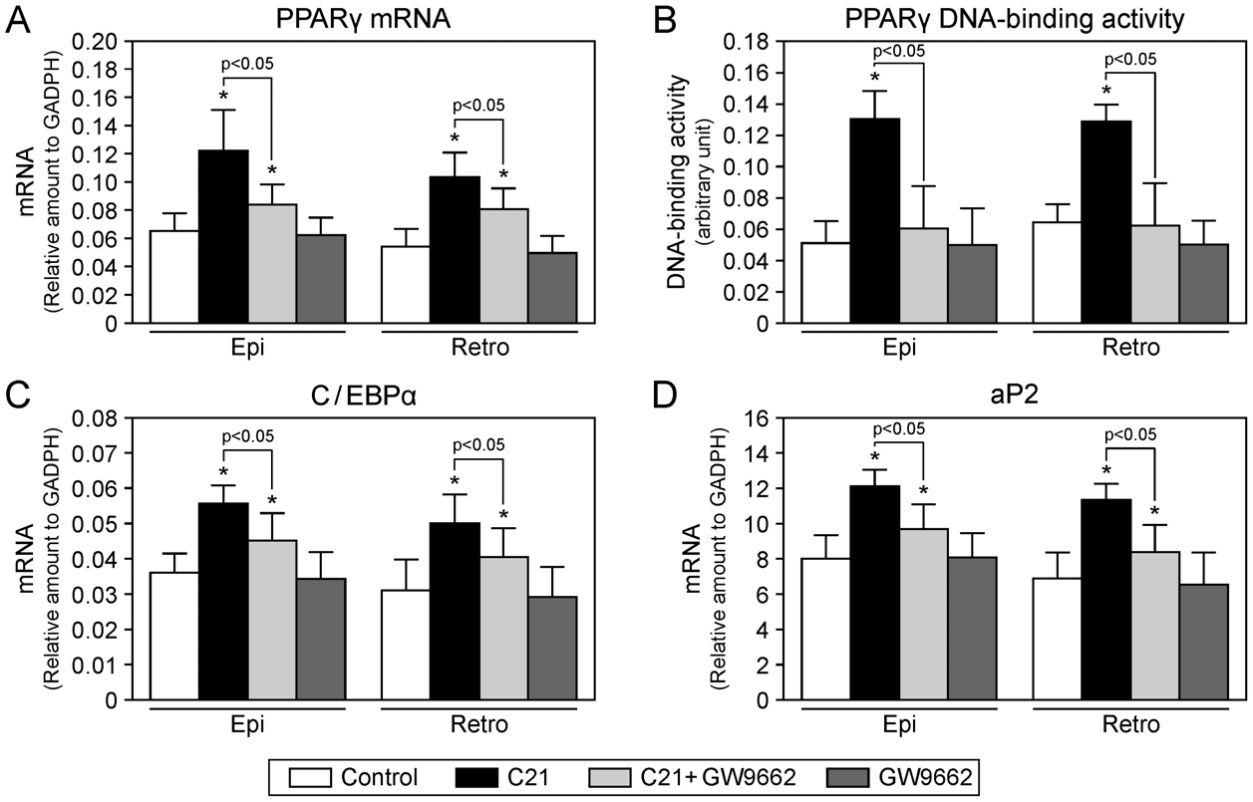
Effects of compound 21 administration on adipocyte differentiation in KK-AY mice. Adipocyte differentiation markers and DNA-binding activity of peroxisome proliferator-activated receptor γ (PPARγ) in epididymal (Epi) and retroperitoneal (Retro) white adipose tissue of KK-Ay mice were determined. mRNA expression (**A**) and in vivo DNA-binding activity of PPARγ (**B**). mRNA expressions of CCAAT-enhancer-binding protein α (C/EBPα) (**C**) and adipocyte Protein 2 (aP2) (**D**). Data are expressed as mean ± SEM. n = 5 to 6 per group. *p<0.05 vs. control. C21, compound 21.

Treatment with C21 increased the mRNA expression of PPARγ in epididymal and retroperitoneal adipose tissue of KK-Ay mice (**Figure 5A**). We next examined DNA-binding activity of PPARγ in these adipose tissues of KK-Ay mice and observed that DNA-binding activity of PPARγ was enhanced in C21-treated mice (**Figure 5B**). These increases in PPARγ expression and DNA-binding activity of PPARγ were inhibited by co-administration of GW9662, whereas treatment with GW9662 did not influence these parameters. The expression of adipocyte differentiation markers, such as C/EBPα and aP2, was increased by C21 treatment (**Figure 5C and 5D**). GW9662 administration attenuated the C21-mediated increase in expression of C/EBPα and aP2, whereas treatment with GW9662 alone did not influence this.

### Effect of compound 21 on expression of adiponectin, inflammatory cytokines, and angiotensin II receptors in white adipose tissue

**Figure 6:**
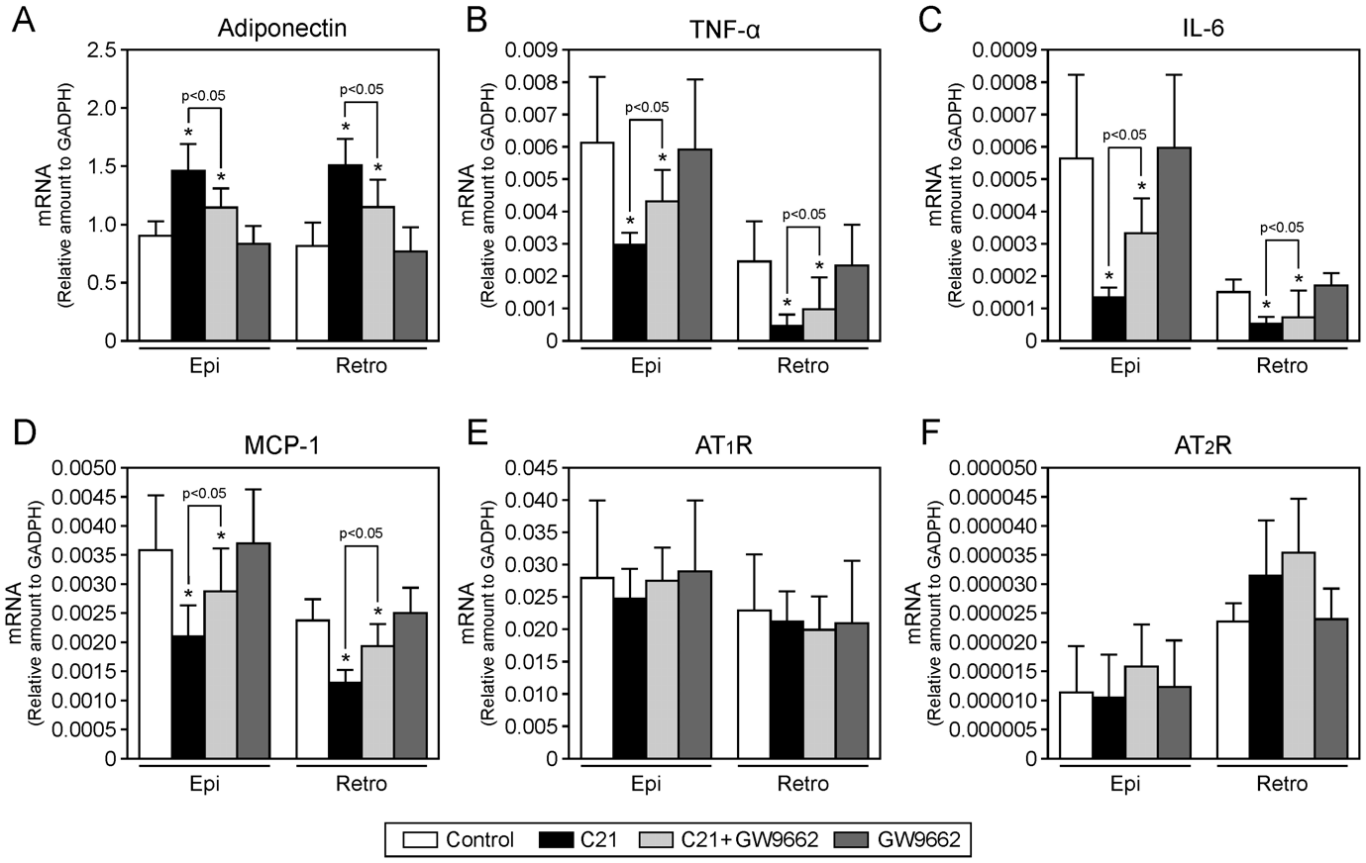
Effects of compound 21 administration on inflammation in KK-Ay mice. mRNA expressions of adiponectin (**A**), tumor necrosis factor-α (TNF-α) (**B**), interleukin-6 (IL-6) (**C**), monocyte chemoattractant protein-1 (MCP-1) (**D**), AT_1_ receptor (**E**) and AT_2_ receptor (**F**) in epididymal (Epi) and retroperitoneal (Retro) white adipose tissue of KK-Ay mice. Data are expressed as mean ± SEM. n = 5 to 6 per group. *p<0.05 vs. control. C21, compound 21.

C21 treatment enhanced mRNA expression of adiponectin in epididymal and retroperitoneal adipose tissue compared with that in control KK-Ay mice, and this increase was attenuated by co-administration of GW9662 (**Figure 6A**). Treatment with C21 decreased mRNA expression of inflammatory cytokines, such as TNF-α, IL-6, and MCP-1, which was inhibited by GW9662 treatment (**Figure 6B, 6C, and 6D**). In contrast, treatment with GW9662 alone did not change mRNA expression of these inflammatory markers. There was no significant difference in mRNA expression of the AT_1_ and AT_2_ receptors in white adipose tissue among the four groups (**Figure 6E and 6F**).

### Recovery of β cell number in pancreas after compound 21 treatment

**Figure 7:**
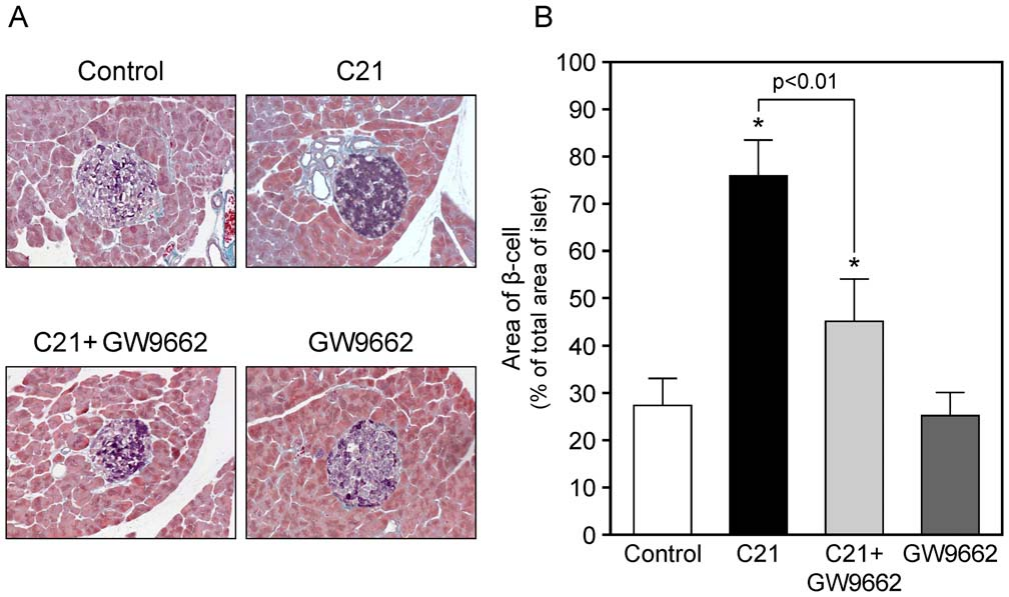
Changes in β cells in pancreatic islets by compound 21 treatment in KK-Ay mice. KK-Ay mice were treated as described in ‘Methods’. The pancreas was taken, fixed with formalin and embedded in paraffin. Sections were subjected to aldehyde-fuchsin staining to detect β cells in pancreatic islets. (**A**) Reproducible microscopic photos of pancreas. Magnification: ×100. (**B**) Quantification of stained β cell area. Area was measured using computer-imaging software as described in ‘Methods’ and expressed as the ratio of β cell area to total islet area. n = 5 to 6 in each group. *p<0.01 vs. control. C21, compound 21.

We observed that C21 treatment increased insulin concentration after a glucose load, and we assumed that C21 could prevent pancreatic β cell damage in KK-Ay. The number of β cells in pancreas islets detected by aldehyde-fuchsin staining was increased in C21-treated mice compared with control and GW9662-treated mice (**Figure 7A and 7B**). This increase in β cell number in the pancreas by C21 treatment was attenuated by GW9662 administration, while treatment with GW9662 alone did not influence these parameters.

## Discussion

The roles of AT_2_ receptor stimulation in the pathogenesis of insulin resistance and functions of adipose tissue in T2DM are still an enigma. Most studies addressing the involvement of the AT_2_ receptor in a variety of experiments have been performed in genetically altered, either AT_2_ receptor-deficient or AT_2_ receptor-overexpressing mice with or without ARB treatment, and unfortunately these experimental approaches have yielded conflicting results. Moreover, PD123319, a widely used AT_2_ receptor antagonist, acts nonselectively at a higher dose [33] and likewise, CGP42112, a widely used AT_2_ receptor agonist, has both agonistic and antagonistic actions depending on its dose used. These findings made it difficult to properly assess AT_2_ receptor actions, especially in vivo. For example, the effect of the AT_2_ receptor on cardiac function after myocardial infarction is controversial, due to lack of an experimental tool to directly stimulate the AT_2_ receptor under in vivo conditions [34]. A novel non-peptide AT_2_ receptor agonist, C21, is a nonpeptidergic agent that has high affinity and selectivity for the AT_2_ receptor [35]. Therefore, it can be expected that C21 will allow the effects of the AT_2_ receptor in cardiac hypertrophy to be studied by means of direct, selective AT_2_ receptor stimulation, which will hopefully help to overcome current controversies [36]. Consistent with this, Kaschina et al reported that treatment with C21 significantly improved systolic and diastolic ventricular function in the rat after myocardial infarction, suggesting that direct AT_2_ receptor stimulation may be a novel therapeutic approach to improve cardiac function after myocardial infarction through anti-apoptotic and anti-inflammatory mechanisms [37]. In keeping with these results, we used C21 to elucidate the roles of direct AT_2_ receptor stimulation in the pathogenesis of insulin resistance in T2DM, mainly focusing on adipose tissue.

We observed that treatment with a non-hypotensive dose of C21 decreased adipose tissue weight/body weight, and that C21 treatment decreased blood glucose and serum insulin concentrations in non-fasting condition in KK-Ay mice. It is well known that insulin resistance is a major metabolic feature of obesity and plays a key role in the etiology of T2DM. We also showed that C21 treatment attenuated the rise of blood glucose concentration after a glucose load, with an increase of serum insulin concentration. Consistent with these results, we demonstrated that C21 treatment increased serum adiponectin and decreased TNF-α concentrations, and enhanced adipocyte differentiation, with increases in mRNA expression of PPARγ, C/EBPα and aP2, and PPARγ activation. These results, including a previous report, [13] suggest that AT_2_ receptor stimulation could enhance adipocyte differentiation, thereby resulting in the improvement of glucose intolerance. However, it should be noted that the lack of hypotensive effects as judged by the tail cuff method does not completely exclude a possible effect of C21 on blood pressure because the tail cuff method does not give accurate readings of diastolic pressure nor does it not provide for 24 hour measurements of blood pressure.

Many lines of evidence have indicated that obesity is closely linked to a chronic inflammatory state, which contributes to metabolic disorders [38]. Adipose tissue is thought to directly exaggerate insulin resistance and to trigger inflammation by secreting adipokines such as IL-6 and TNF-α [1]. Thus, adipose tissue dysfunction can tend toward to a chronic inflammatory state through imbalance of pro-inflammatory and anti-inflammatory activities [39]. Moreover, inflammatory cytokines regulate the differentiation of adipocytes and their function, resulting in worsening of insulin resistance. For instance, TNF-α inhibits adipogenesis by preventing the induction of PPARγ and C/EBPα expression [40]. We observed that C21 treatment increased serum adiponectin concentration and decreased serum TNF-α concentration, and that administration of C21 enhanced mRNA expression of adiponectin and decreased mRNA expression of TNF-α, IL-6 and MCP-1 in white adipose tissue. Moreover, TNF-α is known to directly inhibit insulin signaling, resulting in insulin resistance [41]. Consistent with these findings, we observed that C21 treatment significantly increased 2-[^3^H]DG uptake in white adipose tissue. These results are consistent with the possibility that AT_2_ receptor stimulation by C21 and or some other actions of C21 may attenuate inflammation in white adipose tissue and thereby help enhance adipocyte differentiation and improve insulin resistance. Addressing more detailed mechanisms is important for future clinical application of direct AT_2_ receptor stimulation.

AT_2_ receptor activation is reported to induce PPARγ activation in PC12W cells [26], leading us to hypothesize that AT_2_ receptor-mediated improvement of glucose intolerance is at least in part due to PPARγ activation. We observed that AT_2_ receptor stimulation by C21 enhanced not only PPARγ expression but also DNA-binding activity of PPARγ in white adipose tissue of KK-Ay, suggesting that the possible crosstalk between AT_2_ receptor stimulation and PPARγ activation could be involved in the attenuation of insulin resistance in concert with the regulation of adipocyte differentiation and functions. Accordingly, we demonstrated that C21-mediated improvement of insulin resistance, adipocyte differentiation, and inflammation in adipose tissue were blunted by PPARγ blockade with GW9662, supporting the idea that direct AT_2_ receptor stimulation by C21 ameliorated insulin resistance in T2DM model mice at least partially due to PPARγ activation. In contrast, in adipose tissue, the possibility exists that decreased PPARγ expression improved insulin sensitivity, as demonstrated in PPARγ-deficient mice [42]. Moreover, decreasing PPARγ activity by a specific antagonist was shown to improve insulin sensitivity, accompanied by adipocyte hypotrophy and hyperplasia as observed in PPARγ heterozygous mice [43]. These apparent discrepancies might be dependent on differences in experimental models. It would be intriguing to investigate the roles of possible interaction of the AT_2_ receptor and PPARγ in the pathogenesis of diabetes.

We observed that C21 treatment increased serum insulin concentration in response to a glucose load. Both AT_1_ and AT_2_ receptors are present in various pancreatic tissues, and Ang II has been proposed to have important roles in the pancreas, such as regulating islet β cell insulin biosynthesis and glucose-stimulated insulin release [44]. AT_1_ blockade by ARBs has been reported to have beneficial effects on the pancreas in various diabetes model mice [45]–[47]. However, the role of the AT_2_ receptor in the pancreas is still unclear. Therefore, we examined the possibility that AT_2_ receptor stimulation could protect against β cell damage in the diabetic pancreas and increase insulin production and secretion. We demonstrated that C21 treatment increased the number of β cells in pancreas islets compared with that in control KK-Ay mice. We speculate the reason why the C21 reduced insulin levels in the fed state but increased insulin levels in the OGTT might be due to the preservation of pancreatic β cell number and enhancement of glucose uptake in white adipose tissues, resulting in increases in insulin secretory response and decreases in insulin resistance. The detailed mechanisms of AT_2_ receptor-mediated pancreatic β cell protection in diabetic mice remain to be elucidated. One possible mechanism is a C21-mediated decrease in TNF-α, since TNF-α is known to induce apoptotic cell death in mouse primary β cells and insulinoma cell lines [48], and inhibit glucose-stimulated insulin transcription and secretion observed in the HIT-T15 pancreatic β cell line [49]. We also showed that treatment with GW9662 attenuated the C21-mediated increase of serum insulin concentration after a glucose load and the preservation of β cell number in the pancreas in KK-Ay. Activation of PPARγ has been shown to regulate transcription of several key β cell genes involved in glucose sensing, β cell development, glucose-stimulated insulin secretion, and insulin gene transcription. Activation of these targets leads to enhanced insulin secretion and gene expression in the diabetic state [50]. These data, including our results, suggest that the possible crosstalk between AT_2_ receptor stimulation and PPARγ activation may also be involved in pancreatic β cell function.

It should be acknowledged that the current studies do not prove that any of the metabolic effects of C21 are necessarily mediated by AT_2_ receptor stimulation because we have not excluded the possibility that C21 might be functioning as a direct agonist of PPAR isoforms including PPARγ and PPARα and PPARδ. Future studies will be required to determine the extent to which the metabolic effects of C21 are mediated by its interaction with AT_2_ as opposed to its possible interaction with other receptors such as PPARγ, PPARα, and PPARδ, etc.

Taken together, the current results demonstrate that the AT_2_ agonist C21 improves insulin resistance in T2DM mice in association with enhanced adipocyte differentiation and possibly function, and with protection of β cells in pancreas islets. These effects of C21 were blunted by PPARγ blockade, suggesting a role for PPAR activation in mediating the metabolic actions of C21. The extent to which the metabolic effects of C21 are mediated by its direct interaction with AT_2_ and or by direct interaction with other receptors such as PPARγ, PPARα, and PPARδ remains to be determined. In any case, molecules like C21 may hold promise as potential therapeutic options for the management of insulin resistance and T2DM in the future.
